# Medical News and Miscellany

**Published:** 1898-06

**Authors:** 


### Medical News and Miscellany.

**Dr. G. M. Moody** has removed from Lo to Mexia, Texas.

**For Sale,** very cheap, a Nedofik operating sofa, the same as used by Dr. Wyeth and in the Polyclinic. Address this office.

**Good practice** and drug store for sale cheap. Good reasons for selling out. Address P. R., care Texas Medical Journal, Austin, Texas.

**The students** of the Chattanooga Medical College and others interested will be pleased to know that Dr. E. A. Cobleigh—the original and long time dean of that college, has resumed the deanship.

**Attention** is called to the new advertisement of Lambert Pharmacal Co., "The Treatment of Chronic Diarrhœa." It is a seasonable advertisement. Send for book there offered and mention the Red Back.

**Dr. Fred Hadra,** of San Antonio, son of the distinguished surgeon, Dr. B. E. Hadra, of that place, is surgeon of the First Texas Cavalry (volunteers) U. S. A., with rank of Major. Who says that acquired traits are not hereditary? "Like father, like son," is an old proverb.

**Dr. B. W. McLaughlin,** of Austin, son of Prof. J. W. McLaughlin, M. D., Texas Medical College, passed a No. 1 examination before the Army Board and was commissioned Captain and Assistant Surgeon, and is on duty with First Regiment, Texas Infantry, at Mobile. Are acquired traits transmited? Rather.

**Off for Denver.**—Dr. Hudson, managing editor of the Journal, accompanied by Mrs. H., left on the 3d inst. for Denver via International & Great Northern railroad and Iron Mountain route, to be present at the annual meeting of the American Medical Association, June 7, et seq. (Yours truly has all the work to do this time; our "innings," while ye junior has an "outing.")

**Notice to Our Exchanges.**—Those of our exchanges that have heretofore been sent to Prof. Smith, at Galveston, by our request, should in future be addressed to the TEXAS MEDICAL JOURNAL, Austin, Texas. Please "make a note on it" ("when found").

---

**A New St. Louis Journal.**—There were not enough journals in St. Louis; there is one more now. It is the *St. Louis Medical Gazette*, edited by Dr. M. F. Engman and published by Dr. C. R. H. Davis monthly. We welcome to our exchange list Vol. 1, No. 1, received.

---

**Dr. R. L. Wilson,** of Travis County, a late graduate of the University of Texas, Galveston, has been elected interne at the St. Mary's Hospital, and editor-in-chief of the *Students' Medical Journal.* The doctor favored us with a call lately.

---

**Attention** is called to the new advertisements in this issue, not mentioned elsewhere, to-wit: I. L. Lyons & Co. (Dr. Cock's Antibacilli Compound), the St. Louis Medical College, (Medical Department, Washington University), and the Medical Department of Columbia University, Washington (received after forms were made up).

---

**The Mississippi State Medical Association** has over four hundred active members. Texas, with a population of two and three-quarter millions, and with nearly five thousand physicians—boasts a State Medical Association with three hundred members, after a trial to organize—extending over thirty years! Mississippi has about half the population that Texas has and far less than half as many doctors; perhaps not over a tenth part as many. What's wrong?

---

**Patriotic Doctor.**—Dr. W. W. Walker, of Schulenburg, Texas, a graduate of Tulane University Medical Department, class of 1871, leaves a practice of $3500 to $5000 a year to go to fight the Spaniards. Dr. Walker is an old Confederate soldier. He didn't get enough, it seems, and now he is captain of Troop G, First Texas Calvary, in camp at Austin awaiting orders. We utterly fail to appreciate the doctor's zeal. He has two sons engaged in the practice of medicine, and is several times a grandfather. Just why he wants to "go for a soger"

is best known to himself. Dr. Walker is a long time friend, contributor and subscriber to the Red Back, and our best wishes go with him; but as the Irishman said when the bull butted the locomotive, "I admire your pluck but damn your judgment."

---

TREASURY DEPARTMENT,
OFFICE SUPERVISING SURGEON GENERAL,
MARINE HOSPITAL SERVICE,

WASHINGTON, D. C., June 3, 1898.

A board of officers will be convened at Washington, July 6, 1898, for the purpose of examining candidates for admission to the grade of Assistant Surgeon in the U. S. Marine-Hospital Service.

Candidates must be between twenty-one and thirty years of age, graduates of a respectable medical college, and must furnish testimonials from responsible persons as to character.

The following is the usual order of the examination: 1, physical; 2, written; 3, oral; 4, clinical.

In addition to the physical examination candidates are required to certify that they believe themselves free from any ailment which would disqualify for service in any climate.

The examinations are chiefly in writing, and begin with a short autobiography of the candidate. The remainder of the written exercise consists in examination on the various branches of medicine, surgery and hygiene.

The oral examination includes subjects of preliminary education, history, literature and natural sciences.

The clinical examination is conducted at a hospital, and when practicable candidates are required to perform surgical operations on the cadaver.

Successful candidates will be numbered according to their attainments on examination, and will be commissioned in the same order as vacancies occur.

Upon appointment the young officers are as a rule first assigned to duty at one of the large marine hospitals, as at Boston, New York, New Orleans, Chicago or San Francisco.

After five years service, Assistant Surgeons are entitled to examinations for promotions to the grade of Passed Assistant Surgeon.

Promotion to the grade of Surgeon is made according to seniority, and after due examination as vacancies occur in that grade. Assistant Surgeons receive sixteen hundred dollars, Passed Assistant Surgeons two thousand dollars, and Surgeons twenty-five hundred dollars a year. When quarters are not provided commutation at the rate of thirty, forty or fifty dollars a month, according to grade, is allowed.

All grades above that of Assistant Surgeon receive longevity pay, ten per centum in addition to the regular salary for every five years service up to forty per centum after twenty years service.

The tenure of office is permanent. Officers traveling under orders are allowed actual expenses. For further information, or for invitation to appear before the Board of Examiners, address: SUPERVISING SURGEON GENERAL,
U. S. Marine Hospital Service.

---

### Conclusions as to Antitoxin.

Antitoxin is the most valuable remedy yet devised in the treatment of diphtheria.

Its use is especially valuable in laryngeal cases.

It should be employed at the earliest possible moment after a diagnosis is made.

The initial dose should be (according to the type and severity of the case) from 1000 to 3000 units of a concentrated serum, and should be repeated according to indications.

Local and other general treatment should not be neglected because antitoxin is used.

As a prophylactic agent, its value is unquestioned.

From the information furnished in the reports received, it appears that serum does not, at the present time, offer any advantages over other methods of treatment in tuberculosis.

The number of reported cases in tetanus and puerperal sepsis are too few to justify any conclusions.

ARTHUR JORDAN, M. D., Ch'n,
LANDON B. EDWARDS, M. D.,
E. C. LEVY, M. D.

—*Proceedings Richmond, Va., Academy Medicine.*

