# Erratum

**Published:** 1898-08

**Authors:** 


**Erratum.**—In referring to the new advertisement of The Lambert Pharmacal Co., Choleraic Diarrhœa, in our last issue the printers made us say "chronic diarrhœa" instead of "choleraic diarrhœa."

